# Comparative Transcriptome Analysis Provides Insights Into Yellow Rind Formation and Preliminary Mapping of the *Clyr* (*Yellow Rind*) Gene in Watermelon

**DOI:** 10.3389/fpls.2020.00192

**Published:** 2020-03-11

**Authors:** Dongming Liu, Huihui Yang, Yuxiang Yuan, Huayu Zhu, Minjuan Zhang, Xiaochun Wei, Dongling Sun, Xiaojuan Wang, Shichao Yang, Luming Yang

**Affiliations:** ^1^College of Horticulture, Henan Agricultural University, Zhengzhou, China; ^2^Institute of Horticulture, Henan Academy of Agricultural Sciences, Zhengzhou, China

**Keywords:** watermelon, yellow rind, transcriptome, BSA-seq, gene mapping

## Abstract

As an important appearance trait, the rind color of watermelon fruit affects the commodity value and further determines consumption choices. In this study, a comparative transcriptome analysis was conducted to elucidate the genes and pathways involved in the formation of yellow rind fruit in watermelon using a yellow rind inbred line WT4 and a green rind inbred line WM102. A total of 2,362 differentially expressed genes (DEGs) between WT4 and WM102 at three different stages (0, 7, and 14 DAP) were identified and 9,770 DEGs were obtained by comparing the expression level at 7 DAP and 14 DAP with the former stages of WT4. The function enrichment of DEGs revealed a number of pathways and terms in biological processes, cellular components, and molecular functions that were related to plant pigment metabolism, suggesting that there may be a group of common core genes regulating rind color formation. In addition, next-generation sequencing aided bulked-segregant analysis (BSA-seq) of the yellow rind pool and green rind pool selected from an F_2_ population revealed that the yellow rind gene (*Clyr*) was mapped on the top end of chromosome 4. Based on the BSA-seq analysis result, *Clyr* was further confined to a region of 91.42 kb by linkage analysis using 1,106 F_2_ plants. These results will aid in identifying the key genes and pathways associated with yellow rind formation and elucidating the molecular mechanism of rind color formation in watermelon.

## Introduction

Watermelon (*Citrullus lanatus*) is an important horticultural crop in the Cucurbitaceae family and is one of the top ten most consumed fresh fruits globally. As one of the most popular fruits in many countries, more than 117 million tons of watermelon was produced in 2016 according to the latest statistical data from the FAO (http://www.fao.org). The diploid watermelon has 22 chromosomes (2n = 2x = 22) with an estimated genome size of ~425 Mb (Guo et al., 2013). The draft genome of the East Asia watermelon cultivar 97103 has been sequenced and assembled using NGS sequencing technology (Guo et al., 2013), which greatly facilitated genetic and genomics studies, such as marker development and gene mapping, gene cloning, and genome-wide association analysis (GWAS). Because of the extensive diversity in fruit related traits, such as shape, size, rind thickness and color, flesh texture and color, and content of sugar and carotenoids, watermelons have become one of the model crops for fruit-quality research (Zhu et al., 2016).

Chlorophyll and carotenoids are the main pigments affecting watermelon rind coloration. Because of their essential role in harvesting light energy and converting it into chemical energy, chlorophyll is of great importance in photosynthesis (Fromme et al., 2003). Biosynthesis of chlorophyll belongs to a branch of the tetrapyrrole metabolic pathway (Lange and Ghassemian, 2003) and four distinct sections are included in the biosynthesis progress (Masuda and Fujita, 2008). The first section is synthesis of protoporphyrin IX from 5-aminolevulinic acid (ALA), the precursor of chlorophyll (Hotta et al., 1997). In this progress, ALA is condensed to the monopyrrole, porphobilinogen, and four molecules, and then cyclic tetrapyrrole and uroporphyrinogen III would be synthesized (Grimm, 1998). After decarboxylation and oxidation, protoporphyrin IX is formed at the last step of this section. The second section is the insertion of Mg^2+^ into protoporphyrin IX for Chl a biosynthesis, which is called “the Mg branch” (Walker and Weinstein, 1994). At the last step in the second section, chlorophyllide a would be esterified with a long chain polyisoprenol (geranylgeraniol or phytol) to synthesize Chl a (Tamiaki et al., 2007). The third section is the interconversion of Chl a and Chl b known as “Chl cycle.” In this cycle, a portion of Chl a is converted into Chl b by the activity of Chlide a oxygenase (CAO) (Rüdiger, 2002). Chl b can also be reversibly converted to Chl a (Sundby et al., 1986). The last section is the degradation of Chl a (Takamiya et al., 2000; Hörtensteiner, 2006).

Carotenoids have 40-carbon isoprenoids that play essential roles in light harvesting and photoprotection in photosynthetic organisms, and usually provide characteristic colorations of evolutionary adaptive value in plants, fungi, and animals (Britton et al., 2008; Rebeille and Douce, 2011). More than 750 structurally defined carotenoids have been identified in various organisms including bacteria, archaea, fungi, algae, land plants, and animals (Takaichi, 2011). Chloroplasts of green tissues and chromoplasts of flower petals, fruits, and roots are the main sites where carotenoids are synthesized (Yuan et al., 2015). The carotenoids are initially formed by the synthesis of phytoene *via* geranylgeranyl diphosphate (GGPP) through the innermost isoprenoid pathway (Sundby et al., 1986). Then phytoene is further metabolized through desaturations, cyclizations, and hydroxylations to yield various products, such as lycopene, carotenes, and xanthophylls, by a sequence of tandem reactions (Schofield and Paliyath, 2005).

Varying degrees of yellow and green color have been observed in watermelon rind. According to previous studies, the rind color of many plants in the Cucurbitaceae family is controlled by a single gene. A gene for orange fruit in cucumber and another gene for wax gourd pericarp color were fine mapped, respectively (Li et al., 2013; Jiang et al., 2015). The rind colors of yellow and dark green in watermelon also follow the monogenic inheritance pattern. The gene named *D* for dark green is dominant to the *d* allele for light green rind (Li et al., 2019). One gene named *go* with single recessive inheritance pattern for yellow rind was first reported in 1956 (Barham, 1956). As the fruit matures, color of the fruit rind will change from dark green to golden yellow, and stem and older leaves will become golden yellow (Barham, 1956). Different with gene *go*, another watermelon yellow rind gene following the dominant pattern was mapped to a region on chromosome 4 (Dou et al., 2018). But according to information of the primers sequence and the new released watermelon genome (Watermelon 97103 genome v2) assembled with the PacBio long reads, the dominant gene for yellow rind should be within a region of 729.05 kb but not 59.8 kb (http://cucurbitgenomics.org/organism/21) on the top end of chromosome 4 (Dou et al., 2018).

Recently, watermelon with yellow rind has gained increasing popularity among consumers (Dou et al., 2018), whereas the genes regulating yellow rind and their molecular mechanisms are still unknown in watermelon. In the present study, comprehensive transcriptome analysis for DEG (differentially expressed genes) screening and function prediction between the yellow rind inbred line WT4 and the green rind inbred line WM102 was completed. In addition, with genome resequencing of two parental lines and two DNA pools from the F_2_ population, the *yellow rind* (*Clyr*) gene was mapped to a candidate region on chromosome 4 with F_2_ population plants by BSA-seq and linkage analysis. These results provide new insight into the molecular mechanism of yellow rind formation and aid in elucidating pigment study in watermelon.

## Materials and Methods

### Plant Materials

WM102 is a watermelon inbred line with a dark green rind, which was artificially self-pollinated for at least four generations selected from the Bush Sugar Baby (accession code: Grif15898; provided by USDA-ARS Germplasm Resources Information Network [GRIN] [www.ars-grin.gov]), and the dark green phenotype is stably expressed in this material. WT4 is a yellow rind inbred line, which was used to cross with WM102 to generate F_1_ and F_2_ populations for inheritance analysis and gene mapping. All plants were grown in the greenhouse at the Maozhuang Research Station of Henan Agricultural University (Maozhuang, Zhengzhou, at approximately 113.59°N, 34.87°E). The yellow and green rind phenotype were visually observed and recorded during fruit maturation when the different appearance could be easily distinguished. Segregation ratios of yellow/green rind in the F_2_ population were analyzed with Chi-square tests (χ^2^).

### Chlorophyll and Total Carotenoid Content Determination

**Figure 1:**
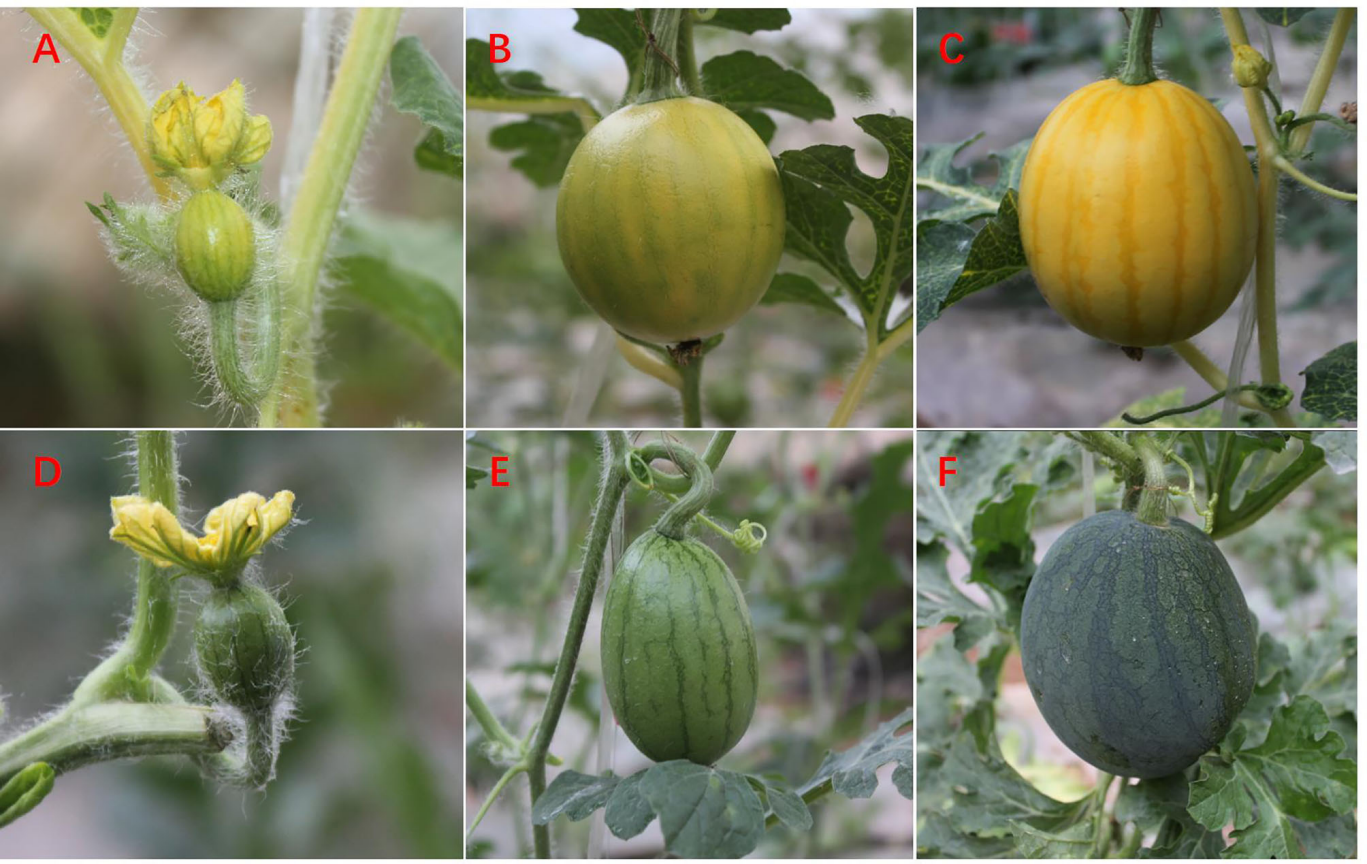
Fruit of watermelon cultivars WT4 and WM102 at critical rind color formation stages. WT4 fruit: 0 DAP **(A)**, 7 DAP **(B)**, and 14 DAP **(C)**. WM102 fruit: 0 DAP **(D)**, 7 DAP **(E)**, and 14 DAP **(F)**.

As rind color of WT4 becomes completely yellow and remains unchanged from 14 days after pollination (**Figure 1**), 1 g sample of fruit rind at 14 DAP was extracted with a mixture of acetone and alcohol (1:1) using a pestle and mortar till residues became colorless. After complete extraction, the absorbance of the extract was read at 663.2, 646.8, and 470 nm on a spectrophotometer (Shimadzu, Kyoto, Japan) and pigment concentrations were calculated according to Lichtenthaler (Harmut, 1987). Each sample was measured with three biological replicates.

### DNA and RNA Extraction

Total genomic DNA from young fresh leaves was extracted using the cetyltrimethylammonium bromide (CTAB) method and the concentration was adjusted to 60 ng/ul (Saghai-Maroof et al., 1984). Fresh samples for RNA extraction were randomly collected from the rinds of three injury-free watermelon fruits in WT4 and WM102 at three different developmental stages (0, 7, and 14 DAP) (**Figure 1**). These samples were immediately frozen in liquid nitrogen, delivered rapidly to the laboratory, and stored at −80– until analysis. For qRT-PCR analysis, total RNA was extracted using the EasyPure^®^ Plant RNA Kit (TRANS) as described by the manufacturer and DNA was removed by digestion with RNase-free DNase. The quality of RNA was assessed with a 1% agarose gel and reverse transcribed to cDNA using a Rever Tra Ace-α-First Strand cDNA synthesis kit (Toyobo).

### RNA-Seq Library Construction, Sequencing, and Reads Mapping

The extracted RNA samples were sent to the Biomarker Technologies Co. Ltd (Beijing) for cDNA library construction. The RNA concentration was measured by NanoDrop 2000 (Thermo) and the integrity was assessed using the RNA Nano 6000 Assay Kit of the Agilent Bioanalyzer 2100 system (Agilent Technologies, CA, USA). After the assessment, mRNA was purified from total RNA using poly-T oligo-attached magnetic beads and eluted in NEB Next First Strand Synthesis Reaction Buffer. After mRNA was fragmented in small pieces under elevated temperature, first strand cDNA was synthesized with a random hexamer primer and M-MuLV Reverse Transcriptase, and the second strand cDNA synthesis was subsequently completed using DNA Polymerase I and RNase H. Remaining overhangs were then converted into blunt ends. The library fragments were purified with the AMPure XP system (Beckman Coulter, Beverly, USA) to select cDNA fragments of preferentially 240 bp in length. Finally, PCR was performed with Phusion High-Fidelity DNA polymerase. Universal PCR primers, Index (X) Primer, and PCR products were purified by the fdAMPure XP system and library quality was assessed with the Agilent Bioanalyzer 2100 system.

After the index-coded samples were clustered on a cBot Cluster Generation System using a TruSeq PE Cluster Kit v4-cBot-HS (Illumia), the library preparations were sequenced on an Illumina HiSeq2000 platform and paired-end reads were generated. The RNA sequences have been deposited in the National Center for Biotechnology Information (NCBI) with the accession number of PRJNA549842. Raw reads were filtered by removing low-quality reads and reads containing the adapter, ploy-N. At the same time, Q20, Q30, GC-content, and sequence duplication level of the clean data were calculated. All downstream analyses were based on clean data with high quality score (Q phred) ≥ 30 (Q30). The clean reads were aligned to watermelon (97103 V1) reference genome sequences released by the Cucurbit Genomics Data Bank (CuGenDB) (http://cucurbitgenomics.org/) using TopHat 2.0.12 (Trapnell et al., 2009). Only reads with a perfect match or mismatches of no more than two bases were further analyzed and annotated based on the reference genome.

### DEGs Screening and Functional Annotation

Expression of three biological replicates was calculated using the DESeq R package to quantify the correlation among biological replicates. DEGs were analyzed with the DESeq2 program based upon reads count (Love et al., 2014). The P-value of DEGs between samples was adjusted using the Benjamini & Hochberg method (Benjamini and Hochberg, 2000). Genes with an adjusted P-value ≤ 0.05 were recognized as DEGs. Three pair-wise comparisons between WT4 (yellow rind) and WM102 (green rind) at three stages (0, 7, and 14 DAP) and comparisons of the WT4 among these three different stages were conducted to identify the genes involved in rind color formation. Gene expression was calculated with well-mapped reads, and the results were normalized to the fragments per kilobase of exon per million mapped fragments (FPKM) with the DESeq2 program (Love et al., 2014). To determine the biological significance of the DEGs, a Gene Ontology (GO) enrichment analysis was implemented using the GOseq R package based Wallenius non-central hyper-geometric distribution (Young et al., 2010). GO terms with a corrected P < 0.05 were considered significantly enriched by DEGs. Similarly, KOBAS software was employed to test the statistical enrichment of DEGs in the KEGG (Kyoto Encyclopedia of Genes and Genomes) database (Kanehisa and Goto, 2000). K-means clustering with 9 times repeated was conducted based on Pearson correlation of gene expression profiles (Walvoort et al., 2010).

### Expression Level Validation of DEGs by qRT-PCR

Real-time quantitative PCR (qRT-PCR) was used to verify the expression results of the selected genes. RT-qPCR was performed with ABI SYBR green in a one-step real-time PCR system according to the manufacturer's instructions. The gene β-actin was used as the internal reference gene to normalize Ct values of each reaction. Each reaction was performed in a final volume of 16 µl, containing 8 µl SYBR Green PCR Master Mix (Applied Biosystems), 250 nM of each primer, and 50 ng cDNA template. The thermal cycling conditions were 94– for 10 min, followed by 40 cycles of 94– for 15 s, 55– for 30s, and 60– for 1 min, with fluorescence detection at the end of each cycle. Amplification of a single product per reaction was confirmed by melting curve analysis. All reactions were performed with three biological replicates. Expression of some genes with significant different expression level according to the RNA-seq result and the genes within the mapping region were analyzed. Sequences of primers for qPCR are listed **Table S1**.

### BSA-Seq Analysis and Preliminary Mapping of Gene *Clyr*

To screen the candidate genomic region responsible for the yellow rind of WT4, 30 yellow-rind plants, and 30 green-rind plants were selected from the F_2_ population for bulking. The total genomic DNA for each plant was exacted and quantified using the NanoDrop 2000 spectrophotometer (Thermo Scientific, USA). Then, two DNA pools were constructed by mixing equal amounts of DNA from 30 yellow-rind (Y-pool) and 30 green-rind plants (G-pool). A 5 ug of sample of DNA from the two bulks and two parental lines were used to construct paired-end sequencing libraries, which were sequenced on an Illumina HiSeqTM 2500 platform.

FastQC was used for cleaning and filtering reads (Andrews, 2010). After low-quality and short reads were filtered out, the remaining high-quality reads of each pool were mapped onto the watermelon reference genome sequence 97103 (ftp://cucurbitgenomics.org/pub/cucurbit/genome/watermelon/97103) by BWA (Li and Durbin, 2009). SNP calling followed GATK Best-Practices (McKenna et al., 2010). First, the MarkDuplicates module was used to mark the duplication alignment. Then the BaseRecalibrator and ApplyBQSR modules were used to detect and correct for patterns of systematic errors in the base quality scores, which act as confidence scores emitted by the sequencer for each base. To ensure the accuracy of SNPs identified by GATK, SAMtools software was also used to detect SNPs. The intersection of SNPs that were detected by both GATK and SAMtools software was designated as final SNPs for further analysis. The obtained SNPs and small indels were noted and predicted using SnpEff software (Cingolani et al., 2012), and only the high-quality SNPs with a minimum sequence read depth of five were used for BSA-seq analysis. The SNP-index is an association analysis method to find the significant differences of genotype frequency between the pools, indicated by Δ(SNP-index), and the detail process was followed as previously (Abe et al., 2012; Takagi et al., 2013). The SNP-index is calculated as follows: SNP‐index (Green) = ρx/(ρX + ρx), SNP‐index (Yellow) = ρx/(ρX + ρx), ΔSNP‐index = SNP‐index (Yellow) − SNP‐index (Green). The Green and Yellow represented the green rind bulk and the yellow rind bulk of the filial generation, respectively. ρX and ρx indicate the number of reads of the alleles in the yellow rind and the green rind parent lines appearing in their pools, respectively. The difference in each locus between the two pools can be observed through the ΔSNP-index. With respect to the qualitative character, the correlation threshold is the theoretical ΔSNP-index value of the corresponding population and the correlation threshold of the F_2_ population is 0.67. The regions over the threshold were considered as the associated candidate regions.

All identified SNPs shared across the bulk were considered polymorphic in association studies and two methods were used to identify the candidate regions associated with yellow rind in watermelon: a Euclidean Distance (ED) algorithm and SNP-Index analysis. The calculation of ED was completed using MMAPPR (Mutation Mapping Analysis Pipeline for Pooled RNA-seq) (Hill et al., 2013) and the high ED value suggested that the SNPs in the genomic regions were closely associated with the targeted genes. Δ (SNP-index) was also used to calculate the association at each SNP position between Y-bulk and G-bulk, and previous detailed processes were followed (Abe et al., 2012; Takagi et al., 2013).

To validate the BSA-seq results and further map the target gene, 30 pairs of SSR markers in the candidate region were selected from a genome-wide SSR development (Zhu et al., 2016) and 30 pairs of Indel primers to screen for polymorphism between WT4 and WM102 (**Table S1**). The 30 pairs of Indel primers between WT4 and WM102 were developed using the newly released watermelon genome (ftp://cucurbitgenomics.org/pub/cucurbit/genome/watermelon/97103/v2/) as reference genome. The markers with good polymorphism were further used to genotype an F_2_ mapping population containing 1,106 plants. PCR amplification of molecular markers and gel electrophoresis were conducted as described in Zhu et al. (2016).

## Results

### Quantification of Chlorophyll and Carotenoids in the Fruit Rind of Two Parental Lines

**Figure 2:**
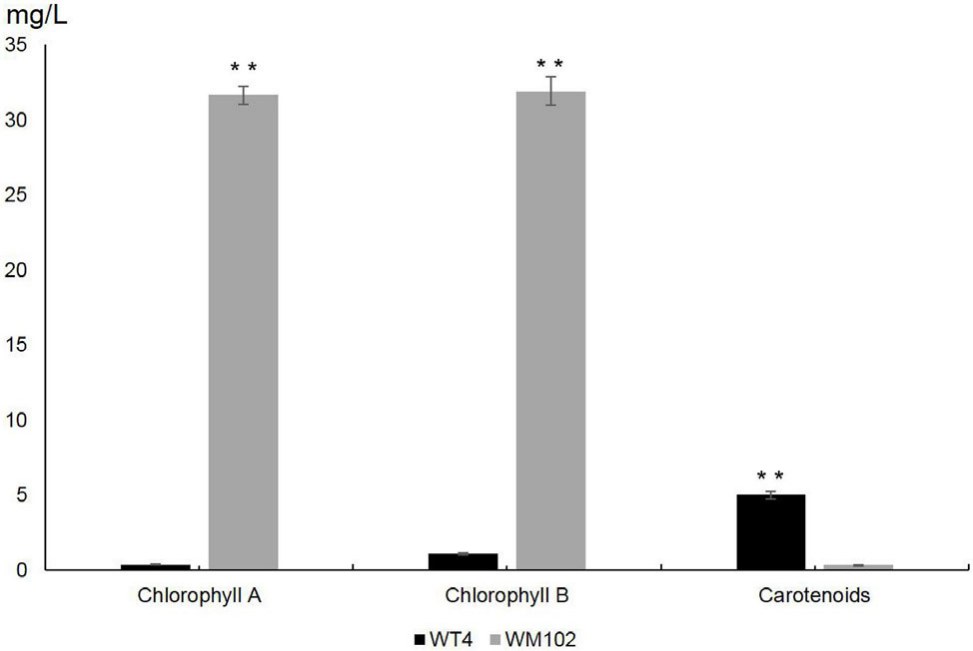
Chlorophylls and carotenoids content in WT4 and WM102 at 14 DAP. Asterisks indicate that the pigment content in WT4 plants was significantly different from that in WM102 plants (**P < 0.01).

To investigate the difference between chlorophyll and carotenoid content in WT4 and WM102, we measured the content of chlorophyll and carotenoid in the fruit rind at 14 DAPs. The content of chlorophyll a and chlorophyll b was significantly reduced in the yellow rind line WT4, which was detected at a very low level, whereas the carotenoids were dramatically increased in WT4 at the same developmental stage (**Figure 2**). The chlorophyll in the green rind line WM102 was at a much higher level compared with that of WT4, and the carotenoids were almost undetectable. This indicated the formation of yellow rind in WT4 was probably because of the reduced chlorophyll and increased carotenoids.

### RNA-Seq and Transcript Assembly Identify Novel Genes

To investigate the transcriptomic difference between WT4 and WM102, a total of 18 cDNA libraries were constructed and sequenced for three developmental stages during rind color formation at 0, 7, and 14 DAP for two parental lines. Approximately 124.71 Gb clean reads were obtained for the 18 cDNA libraries, ranging from 5.96 to 7.98 Gb reads per library. All the clean reads were deposited in the NCBI Short Read Archive (SRA) database under number PRJNA549842.

**Figure 3:**
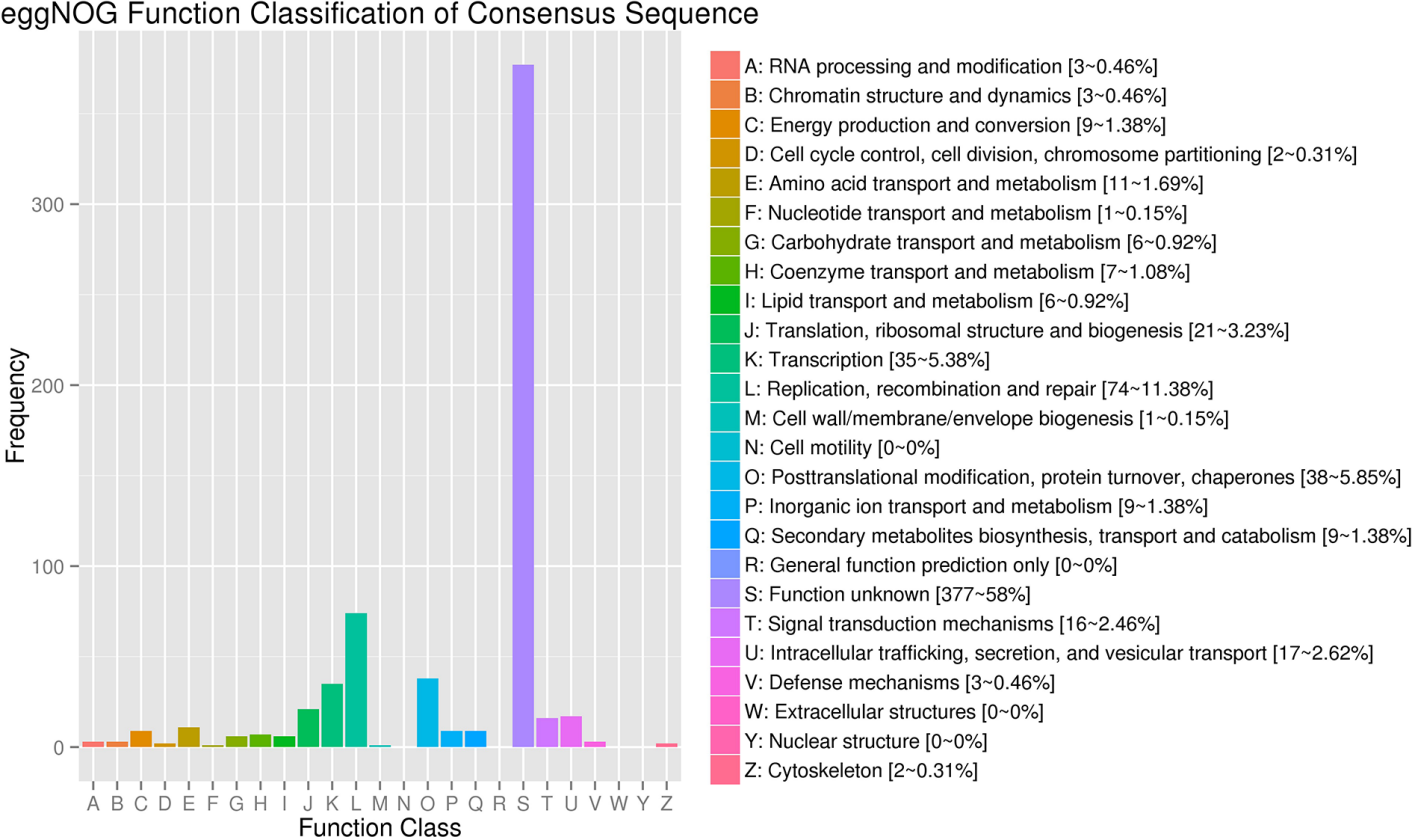
eggNOG Function Classification of novel genes.

The clean reads of each sample were separately mapped to the watermelon reference genome 97103 with a mapping rate ranging from 78.31 to 95.83% (**Table S2**). Compared with 23,440 predicted genes in the previous annotations of the watermelon genome, a total of 24,805 genes were identified in the assembly of 18 transcriptomes including 1,365 novel isoforms of unknown genes detected in our study (**Table S3**). The 24,805 genes were used as reference transcripts to determine the read count with HTSeq (Anders et al., 2015). The 1,365 novel genes were further functionally annotated by aligning the sequence to the NCBI non-redundant (Nr) (Pruitt et al., 2005), SwissProt (Boeckmann et al., 2003), GO (Harris et al., 2004), Pfam (Finn et al., 2013), and KEGG (Kanehisa and Goto, 2000) protein databases (e-value <1e-5) by BLAST software. As a result, 818, 429, 433, 387, and 217 genes were successfully annotated in the five above protein databases, respectively (**Table S4**). In addition to the genes whose function is unknown, most of the novel genes were related to “Replication, recombination, and repair,” “Transcription,” “Translation, ribosomal structure, and biogenesis,” “Defense mechanisms,” and “Extracellular structures” (**Figure 3**).

**Figure 4:**
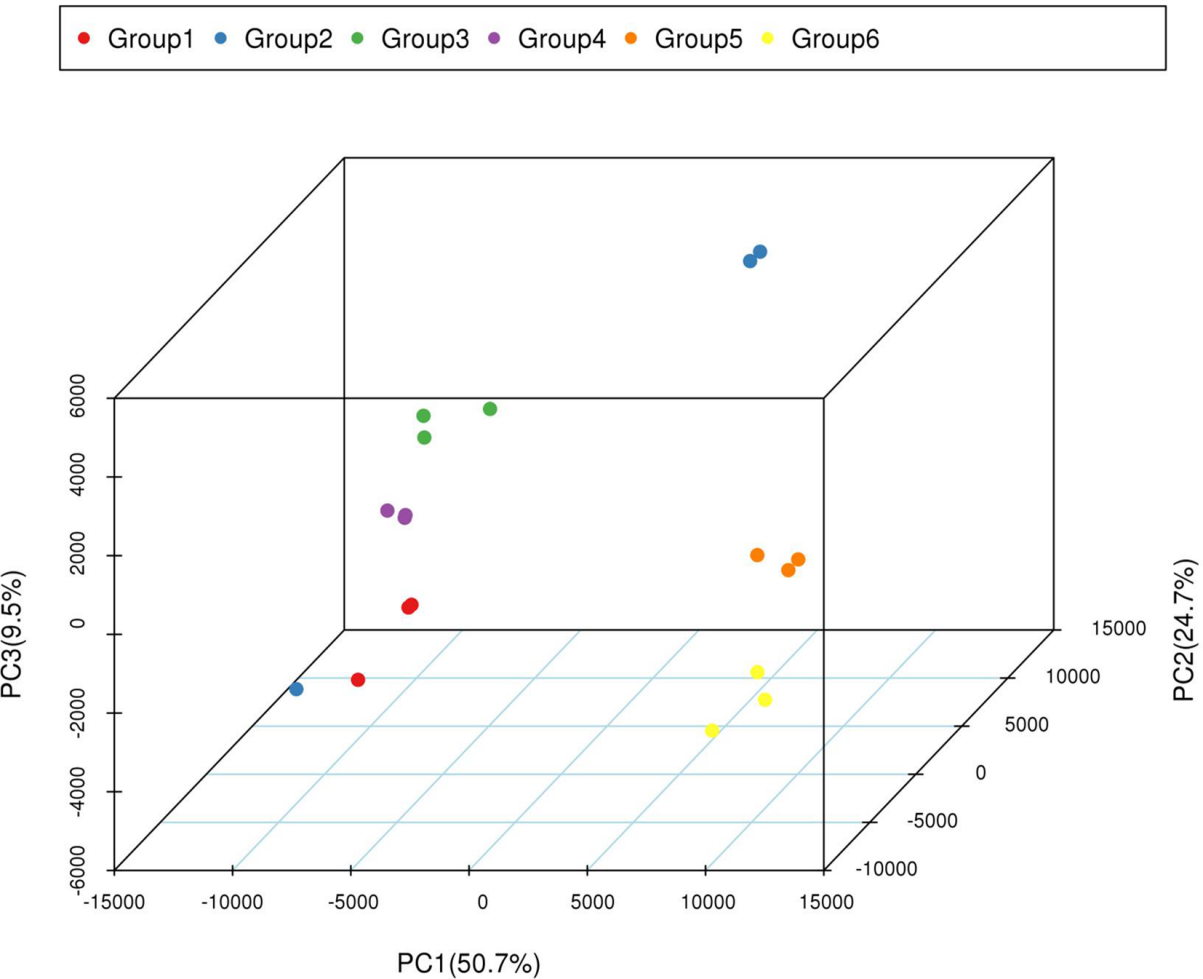
PCA of transcriptomic dataset. Since each sample was repeated three times, Group 1, 3, and 5 contains the repeated samples of WT4 at 0 DAP, 7 DAP, and 14 DAP; Group 2, 3, and 6 contains the repeated samples of WM102 at 0 DAP, 7 DAP, and 14 DAP.

The correlation coefficients among three replicates of each period ranged from 0.91 to 0.99, except the coefficients in group 2 (**Table S5**), indicating that most of the three replicates were consistent. The PCA (principal component analysis) analysis indicated that most of the variation in gene expression among different plants was a consequence of the developmental stage. Furthermore, six distinct groups formed within each group, indicated that the transcriptomes of the yellow-rind and green-rind clearly differed from each other (**Figure 4**).

### DEGs Analysis of WT4 and WM102 During Fruit Rind Coloring

**Figure 5:**
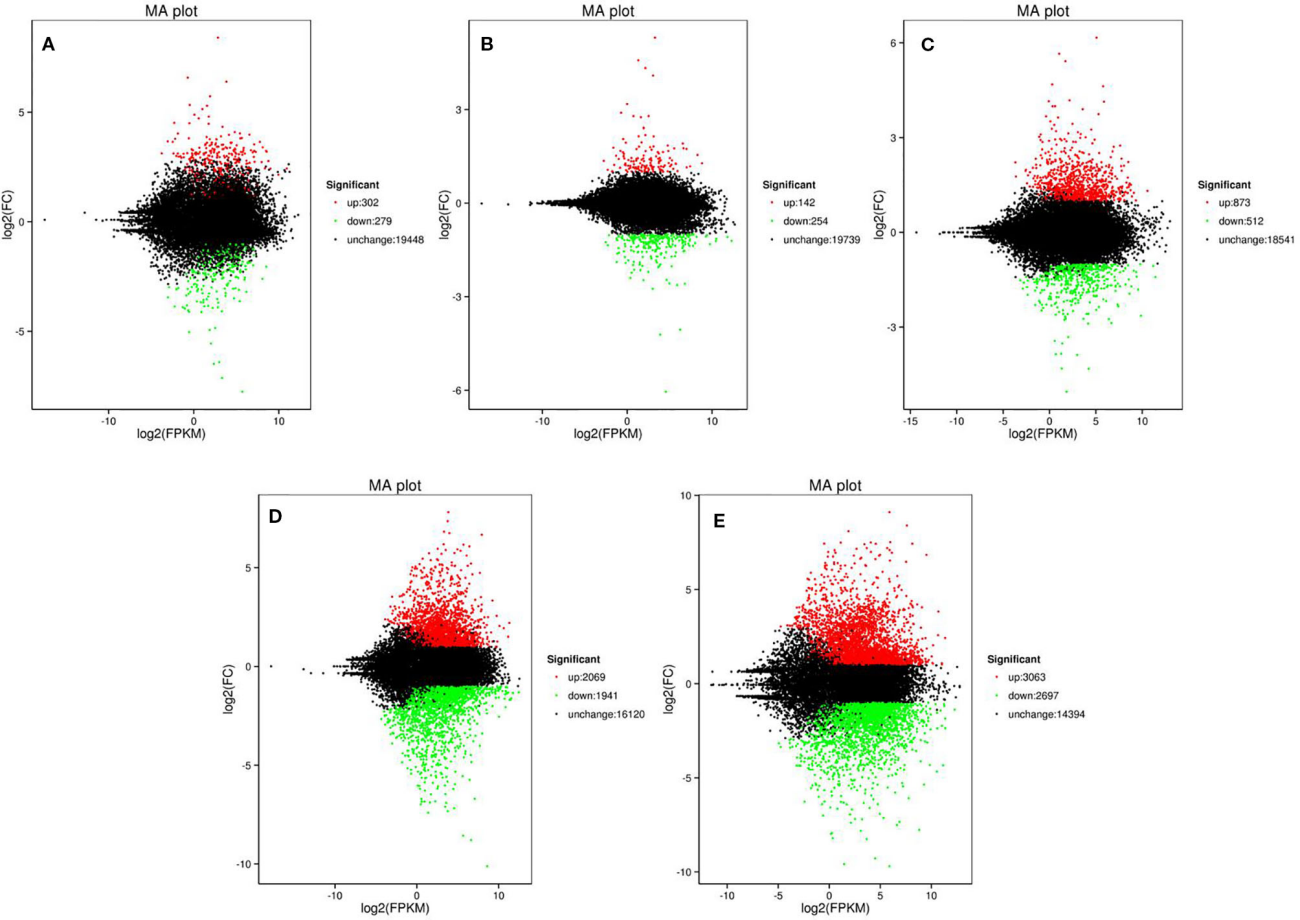
Volcano plot showing DEGs between different libraries. P < 0.05 was used as the threshold to judge the significance of difference in gene expression. Red plots represent up-regulated genes; green plots represent down-regulated genes; black plots represent genes with no significant difference. **(A–C)** present genes at 0 DAP, 7 DAP, and 14 DAP between WT4 and WM102; **(D, E)** present genes of WT4 at 7 and 14 DAP compared with 0 and 7 DAP, respectively.

To screen the genes affecting rind color formation, a stringent value of FDR ≤ 0.05 and an absolute value of log2 Ratio ≥ 2 was used as the thresholds to identify the DEGs between WT4 and WM102 at the three stages (0, 7, and 14 DAP). The DEGs between two close stages during fruit rind coloring of WT4 (the earlier stage was considered the control sample, and the later stage was the treated sample). At 0 DAP, the color of WT4 is green-yellow, whereas it is green in WM102. Correspondingly, 581 DEGs between WT4 and WM102 were obtained. Among the 581 DEGs, 302 genes were up-regulated and 279 genes were down-regulated in WT4 (**Figure 5A**). At 7 DAP, the color of WT4 was yellow but it was still green in WM102. Correspondingly, the number of DEGs between WT4 and WM102 was 396, 142 genes, and 254 genes that were up-regulated and down-regulated in WT4 (**Figure 5B**). At 14 DAP, the color of WT4 changed to golden yellow and it is dark green in WM102. A total of 1,385 DEGs were obtained through screening. Among the 1,385 DEGs, 873 were up-regulated and 512 were down-regulated (**Figure 5C**). Because the color of WT4 obviously changed at the three stages, the gene expression level of WT4 at different stages was analyzed. Compared with the genes at 0 DAP, 4,010 DEGs were obtained at 7 DAP in WT4, 2,069 were up-regulated and 1,941 were down-regulated (**Figure 5D**). At 14 DAP, 5,760 DEGs were compared with the genes at 7 DAP, 3,063 were up-regulated and 2,697 were down-regulated (**Figure 5E**). The number of DEGs of WT4 at different stages is much more than that of WT4 and WM102 at the same periods.

**Figure 6:**
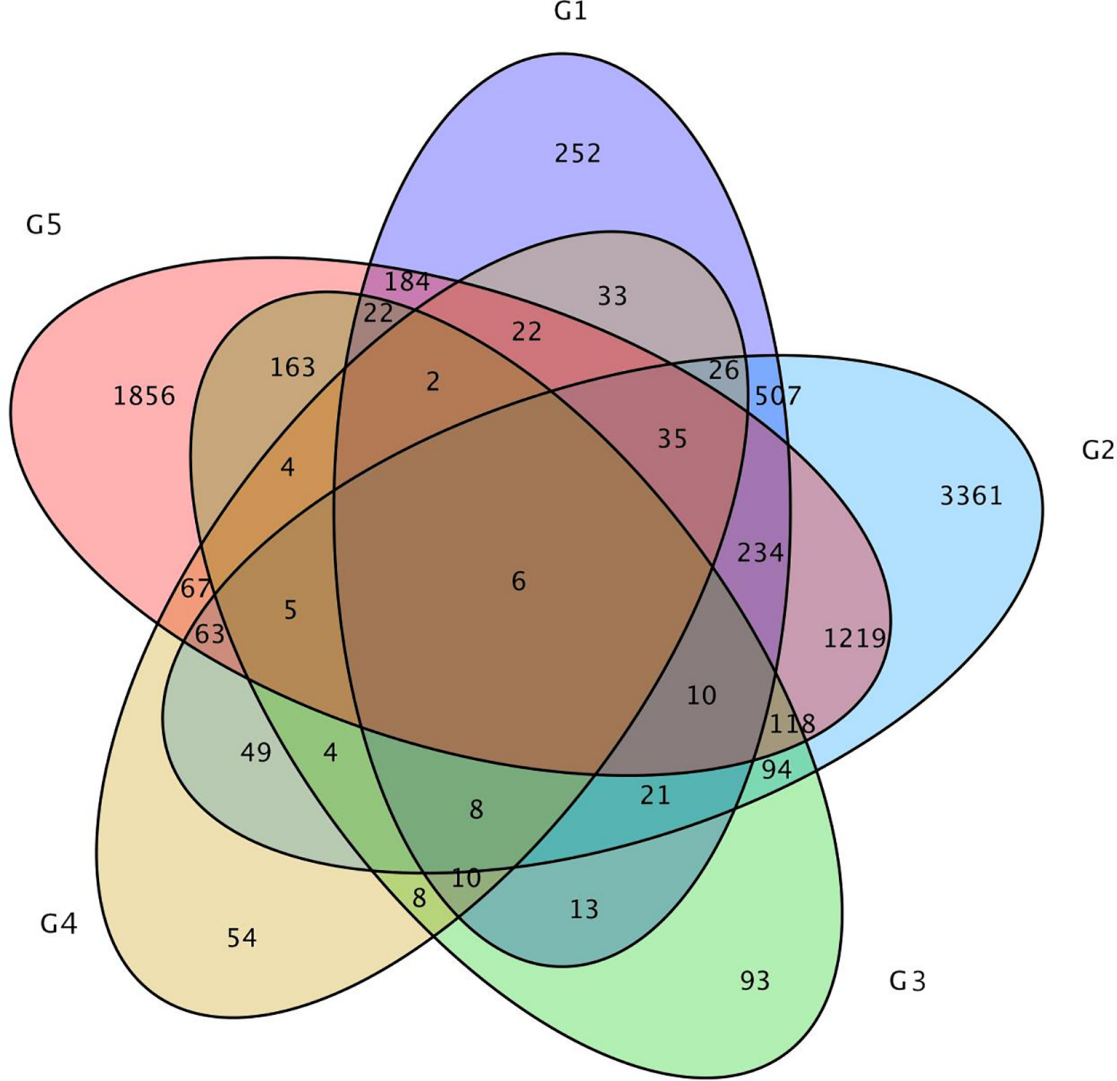
Venn diagram of the relationship between DEG groups. The numbers indicate the DEG number in each DEG group. Group 1, 2, and 3 contains DEGs at 0, 7, 14 DAP between WT4 and WM102, respectively. Group 4 and 5 contains DEGs at 7 and 14 DAP compared with 0 and 7 DAP of WT4, respectively.

In addition, only 6 DEGs were shared by the above five DEGs groups. Approximately 47 DEGs were shared by the 0-DAP and 7-DAP groups, 142 DEGs were shared by 7-DAP and 14-DAP groups, and 92 DEGs were shared by 0-DAP and 14-DAP groups (**Figure 6**). This further suggested that a common group of genes was activated or deactivated concerning fruit rind coloring.

### Identification of DEGs Expression Patterns

**Figure 7:**
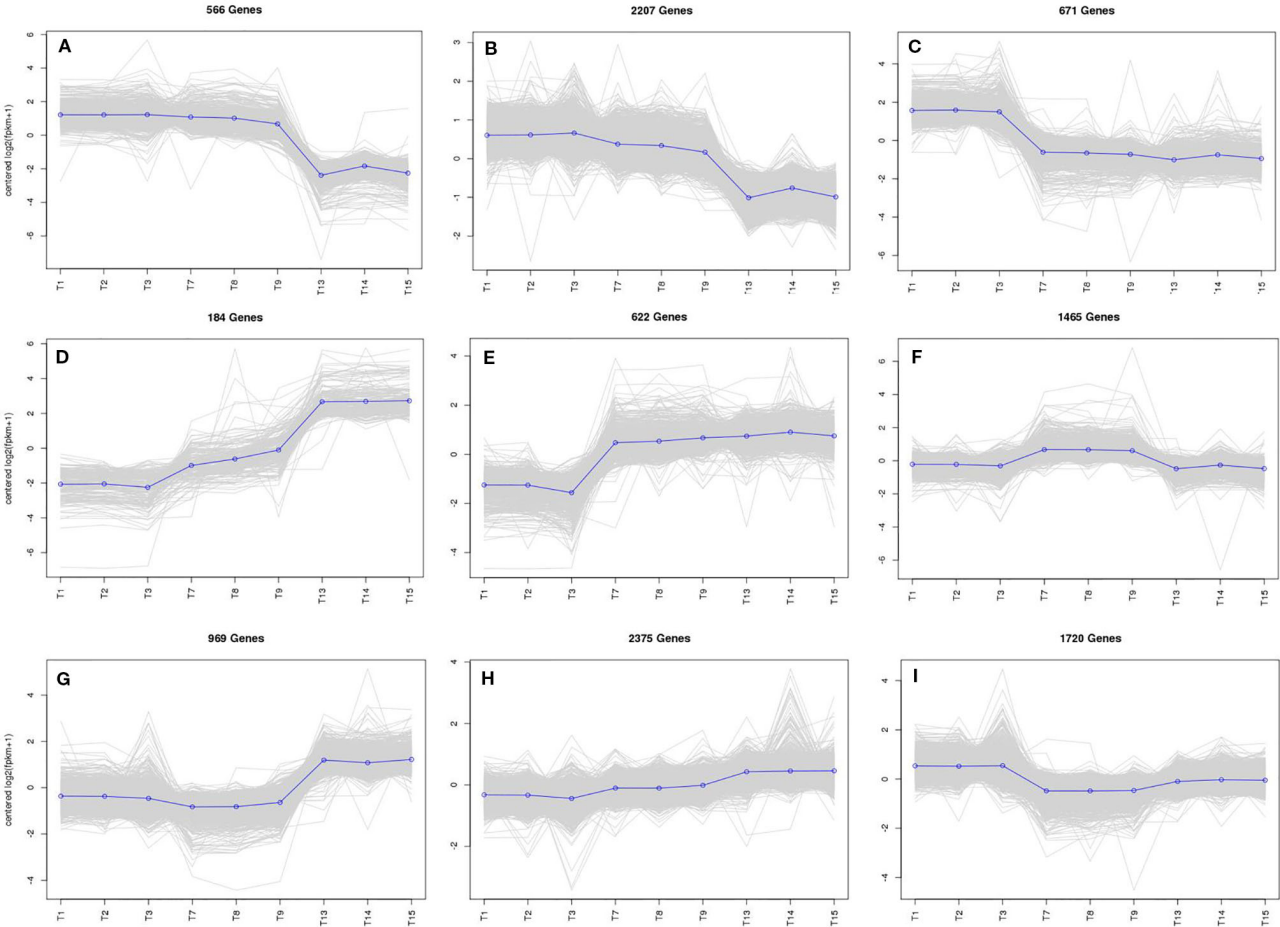
The main clustering of DEGs expression patterns. T1, T2, and T3 in each picture are the gene expression levels of the three repetitions at 0 DAP of WT4; T4, T5, and T6 in each picture are the gene expression levels of the three repetitions at 7 DAP of WT4; T7, T8, and T9 in each picture are the gene expression levels of the three repetitions at 14 DAP of WT4. **(A–I)** present the different expression patterns of the relative expression level of DEGs in WT4.

To study the DEG expression patterns, the relative expression level of DEGs in WT4 were analyzed by K-means clustering algorithm (Hartigan and Wong, 1979). Results show that there mainly existed nine DEGs expression patterns (subclusters) in WT4 (**Figure 7**). The most prominent group was subcluster_8, in which 2,375 genes were expressed with slight increase levels from stage 1 to stage 3 (**Figure 7H**). In subcluster_6, 1,465 genes were expressed with a slight higher level at 7 DAP than that at both 0 DAP and 14 DAP (**Figure 7F**). In subcluster_7, 969 genes were significantly expression-upregulated at 14 DAP compared with 7 DAP, but with no obvious expression level changes at 7 DAP compared with 0 DAP (**Figure 7G**). A similar pattern was observed for subcluster_4, where genes revealed a significant higher expression level at 14 DAP compared with 0 DAP but a light higher expression level at 7 DAP compared with 0 DAP (**Figure 7D**). Different from subcluster_4 and subcluster_7, 622 genes in subcluster_5 were significantly up-regulated at 7 DAP and 14 DAP compared with expression level at 0 DAP (**Figure 7E**). Contrary to subcluster_5, 671 genes in subcluster_3 were down-regulated at 7 and 14 DAP (**Figure 7C**). A similar expression pattern was exhibited in subcluster_9, but the expression level recovered to a light higher level at 14 DAP (**Figure 7I**). Genes in subcluster_1 were down-regulated at 14 DAP, but the expression level at 7 DAP was similar to that at 0 DAP (**Figure 7A**). Similarly, in subcluster_2, 2,207 genes were significantly down-regulated at 14 DAP, the expression level at 7 DAP was slightly lower than that at 0 DAP, but it was higher than that at 14 DAP (**Figure 7B**). These dynamic gene expression patterns further suggest that yellow rind color is formed *via* a highly complex process.

### GO Term Enrichment Analysis of DEGs

To further characterize the function of the DEGs, GO enrichment analysis was completed with GOseq. The top 10 enrichment terms in biological process, cellular component, and molecular function of 0, 7, and 14 DAP were selected as the main nodes of the directed acyclic graph, respectively (**Table S6**). Of all these enrichment terms, some regarding the metabolism and function of chlorophyll and carotenoids were identified as significant ones. At 0 DAP, the chloroplast membrane was identified as one of the important terms in cellular components, and the gene *Cla011368* (*Cla97C01G001920*) belonging to chloroplast membrane term participates the chlorophyll biosynthetic process and protochlorophyllide reductase activity according to the watermelon genome (**Table S6**). At 7 DAP, the important term chloroplast photosystem II was identified among the enrichment terms in biological process, cellular component, and molecular function (**Table S6**). The chloroplast photosystem II is composed of an inner complex, which contains five chlorophyll a molecules, which have an inner antenna function (Bassi and Dainese, 1992). Genes *Cla001715* (*Cla97C05G096030*) and *Cla021166* (*Cla97C05G081100*) in this term contains the photosystem II oxygen domain and belong to the oxygen evolving enhancer protein 3 family. Except the two important terms above, some other terms, such as photosystem I, plasma membrane, chloroplast inner membrane, light harvesting, photosynthesis, and chlorophyll binding are also recognized (**Table S6**).

### KEGG Pathway Enrichment Analysis of DEGs

**Figure 8:**
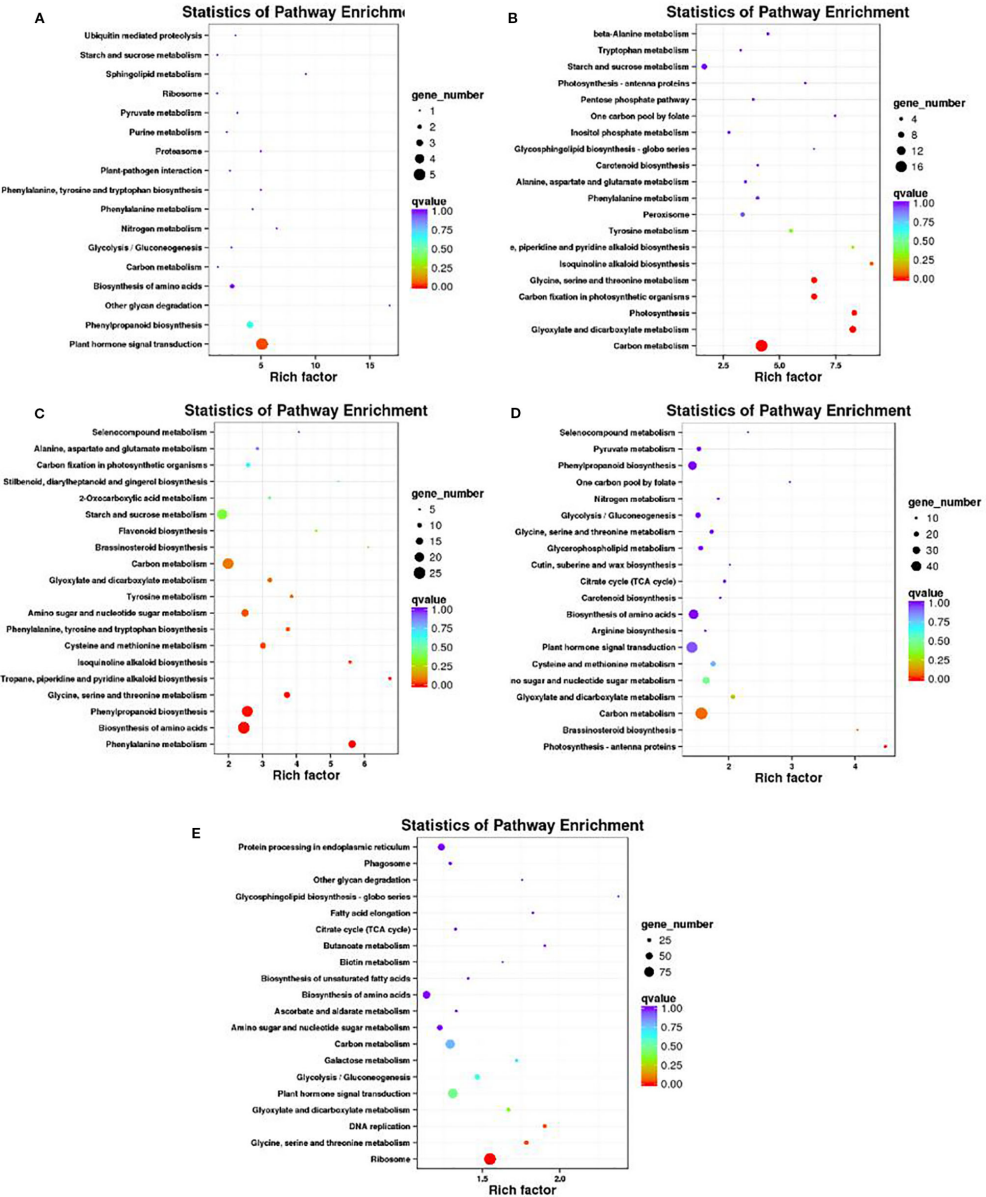
Scatter plot of the KEGG pathway enrichment of DEGs. Rich factor is the ratio of the DEG number to the background number in a certain pathway. The size of the dots represents the number of genes, and the color of the dots represents the range of the q-value. **(A–C)** present the significant terms at 0 DAP, 7 DAP, and 14 DAP between WT4 and WM102; **(D, E)** present the significant terms of WT4 at 7 and 14 DAP compared with 0 and 7 DAP, respectively.

A KEGG pathway enrichment analysis of DEGs was conducted to identify the biological pathways of incompatible interaction. The 20 top KEGG pathways with the most representation are shown in **Figure 8**. At 0 DAP, the gene number of plant hormone signal transduction and phenylpropanoid biosynthesis were significantly higher than that of the other pathways (**Figure 8A**). As a major component of plant specialized metabolism, phenylpropanoid biosynthetic pathways provide anthocyanins for pigmentation, flavonoids (Ferrer et al., 2008). The pathways with most genes at 7 DAP are for carbon metabolism, glyoxylate, and dicarboxylate metabolism, photosynthesis, and carbon fixation in photosynthetic organisms (**Figure 8B**). The pathways, such as glyoxylate and dicarboxylate metabolism, photosynthesis, and carbon fixation in photosynthetic organisms were closely related to chlorophyll metabolism. Similar to 0 DAP, the phenylpropanoid biosynthesis pathway, which is concerned with the anthocyanins for pigmentation and flavonoids biosynthesis at 14 DAP contained many DEGs (**Figure 8C**). Compared with the former stage, the KEGG pathways at 7 DAP and 14 DAP are mainly focused on carbon metabolism and plant hormone signal transduction. The phenylpropanoid biosynthesis pathway was also enriched at 7 DAP (**Figure 8D**). Differently, the pathways, including protein processing in the endoplasmic reticulum and ribosomes also contained many more genes than other pathways (**Figure 8E**).

**Figure 9:**
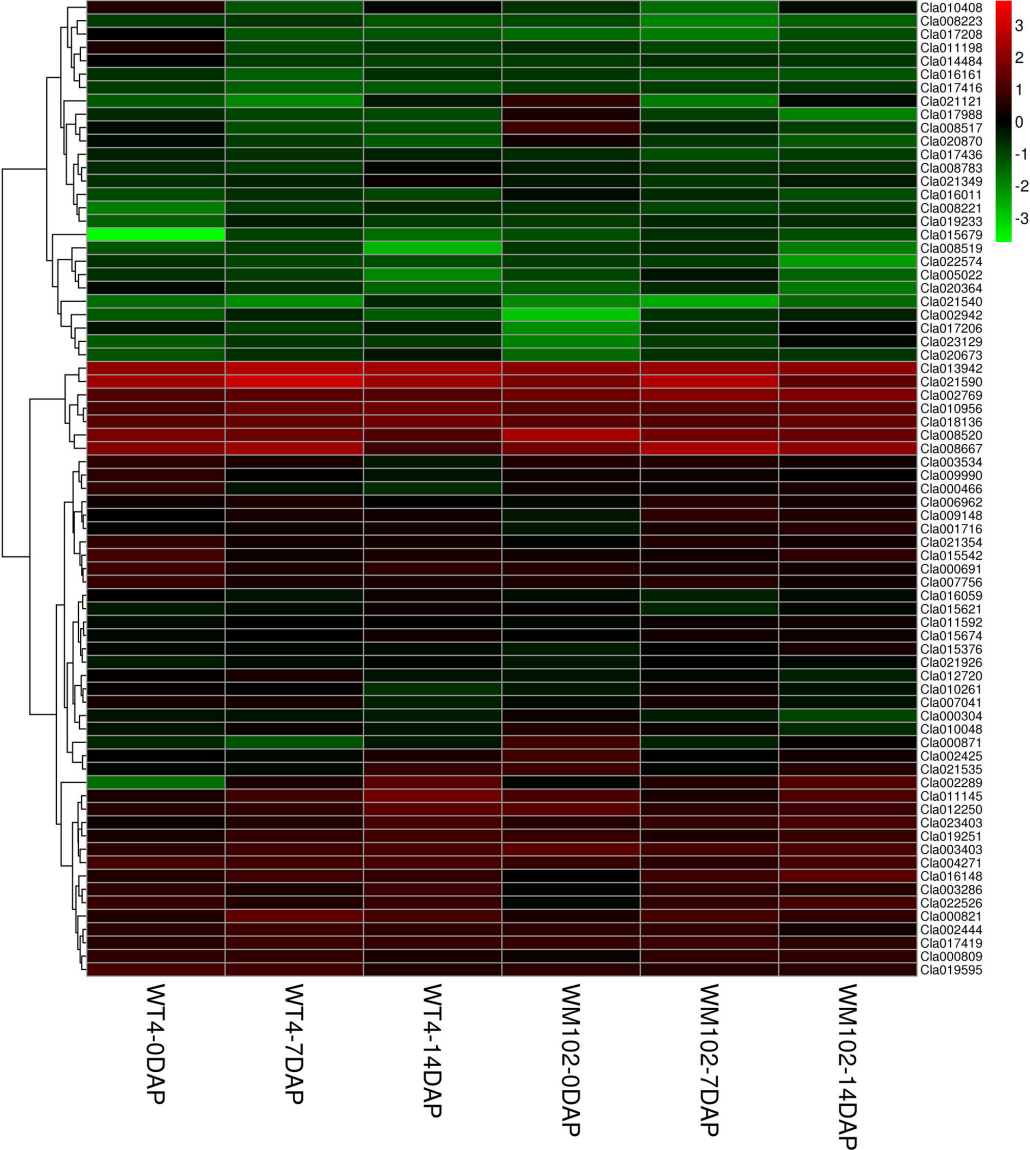
DEGs involved in chlorophyll and carotenoid metabolism during the ripening of WT4 and WM102 fruit rind.

Because metabolism becomes increasingly active in fruit growing and ripening at 7 DAP and 14 DAP, many DEGs are related with other characters. The chlorophyll a and chlorophyll b content in the WT4 rind was much lower than that of WM102 in the maturation period, whereas the carotenoid content in WT4 was much higher than that of WM102. A total of 56 and 9 DEGs concerning chlorophyll and carotenoid metabolism between WT4 and WM102 at the three stages were obtained (**Figure 9**). Many of these DEGs play important roles in plants chlorophyll and carotenoid biosynthesis. For example, gene *Cla013942* (*Cla97C08G148420*) codes for the Photosystem II Protein.

Photosystem II is the multi-component enzyme of cyanobacteria, algae, and plants that catalyze the light-driven oxidation of water to molecular oxygen. This complex consists of more than 20 proteins, including both integral membrane and extrinsically associated proteins (Roose et al., 2007). Gene *Cla008566* (*Cla97C02G049100*) is responsible for coding of magnesium chelatase subunit D. Magnesium chelatase inserts Mg^2+^ into protoporphyrin IX in the chlorophyll and bacteriochlorophyll biosynthetic pathways, which is the key step during chlorophyll a biosynthesis (Willows and Beale, 1998). The chlorophyll and carotenoid mechanisms are very complex, concerning multiple metabolic activities, abnormal expression of each gene in the process may affect pigment biosynthesis and rind color formation.

**Figure 10:**
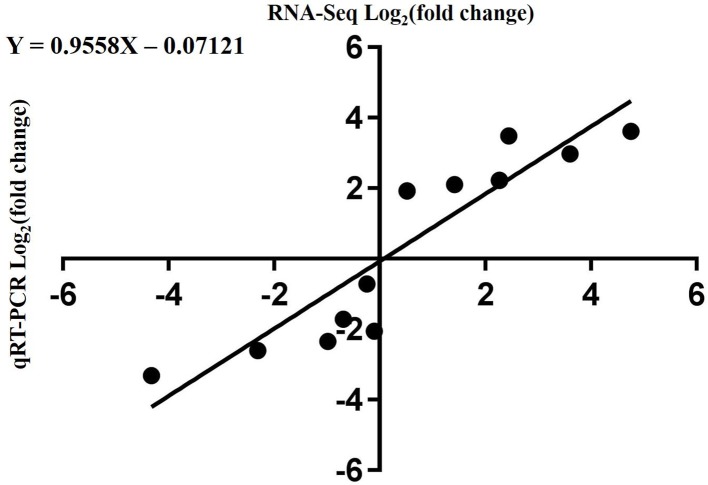
Correlation of expression levels between RNA-Seq and qRT-PCR.

To validate the RNA-Seq data, qRT-PCR was performed for 13 DEGs identified by RNA-seq analysis. The 13 genes were selected to reflect some of the functional categories and pathways related to chlorophyll and carotenoid biosynthesis. Comparison with the RNA-Seq data showed that the trends in these gene expression patterns were consistent and had a strong positive correlation coefficient (R^2^ = 0.9558), indicating that the DEGs detected from RNA-Seq analysis were reliable (**Figure 10**).

### Mapping of *Clyr* Gene by BSA-Seq Analysis and Linkage Analysis

**Figure 11:**
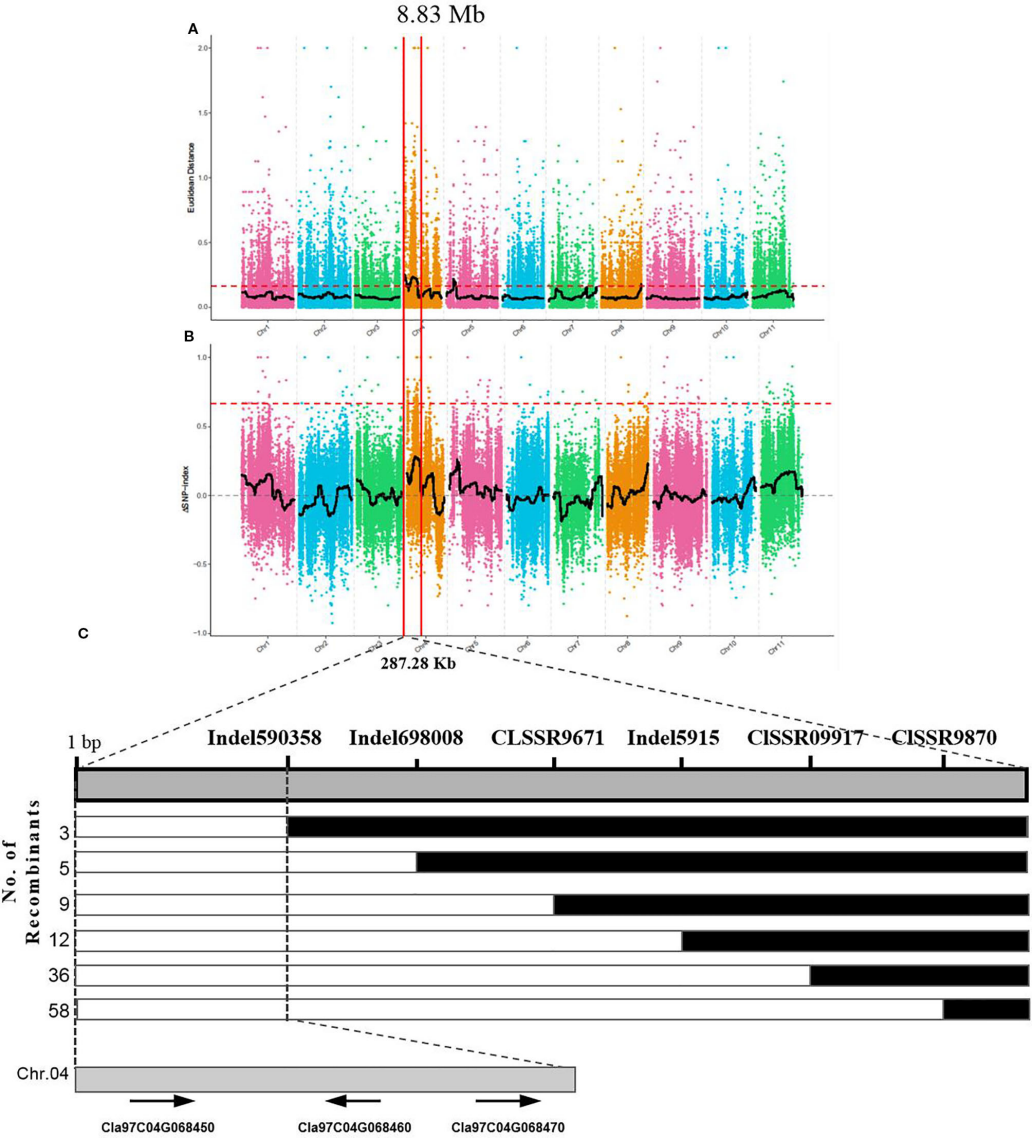
BSA-seq analysis and preliminary mapping of the candidate regions by linkage analysis. BSA-seq analysis using Euclidean Distance (ED) algorithm **(A)** and SNP-Index method **(B)**. The candidate gene was confined to a region of 228.7 kb on the top end of chromosome 4 **(C)**. The colored dots represented the calculated SNP-index (or ΔSNP-index) value, and the black line is the fitted SNP-index (or ΔSNP-index) value. The red dashed line represents the significant threshold.

The yellow rind phenotype in the F_2_ population could be easily identified after fruit maturation, and a total of 1,106 plants of the F_2_ population were investigated in terms of rind color. Among them, 818 plants were observed with yellow rind, and 288 of them were green rind plants. This was consistent with a 3 to 1 segregation ratio (P = 0.583 in a χ^2^ test against 3:1). These results suggested that the *yellow rind* (*Clyr*) in watermelon was controlled by a dominant gene. To explore further the candidate gene regulating yellow rind formation in WT4, a BSA-seq strategy was used to identify the candidate region harboring the *Clyr* gene. We randomly selected 30 yellow rind plants and 30 green rind plants from the F_2_ population to mix the Y-bulk and G-bulk for NGS sequencing. After filtering low-quality reads, the resequencing of the two parental lines generated 79,907,647 and 73,483,217 clean reads with 23.94 and 22.02 Gb for WT4 and WM102, respectively. For the two bulks, a total of 30.97 Gb clean data were obtained (15.43 Gb for the Y-bulk and 15.43 Gb for the G-bulk) with an average depth of 29 × the genome assembly. There were 111,074 high-quality SNPs detected after filtering SNPs with low coverage and discrepancy between parental lines and bulks. To identify the genomic region associated with yellow rind phenotype, we used the ED algorithm and the SNP-Index to measure the allele segregation of SNPs between the two bulks. In the SNP-index analysis, there was no significant region identified associated with the yellow rind trait. However, there was an obvious peak under the significant threshold which was located in the same candidate region detected by ED analysis. The most significant region associated with yellow rind detected by ED algorithm was on watermelon chromosome 4 from 0 to 8.83 Mb (**Figure 11A**), and the candidate region detected by the SNP-Index was from 4.63 to 7.77 Mb of chromosome 4 (**Figure 11B**).

**Figure 12:**
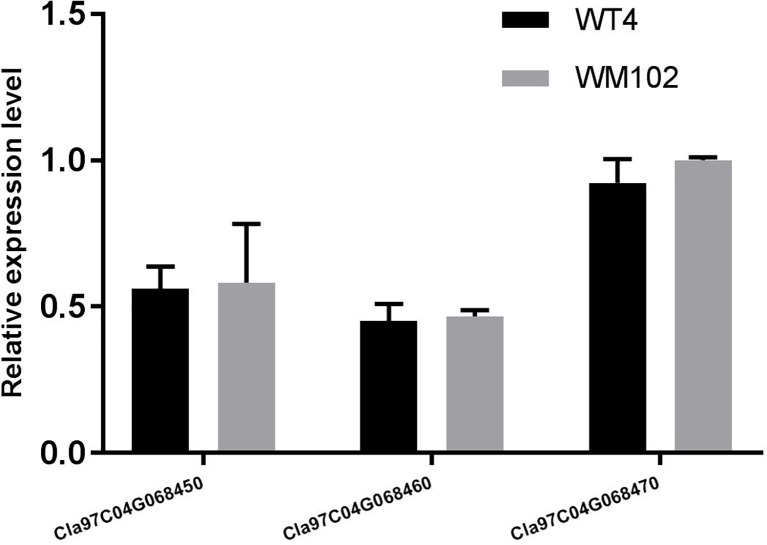
Relative expression level of genes *Cla97C04G068450* (*Cla002781*), *Cla97C04G068460* (*Cla002779*), and *Cla97C04G068470* (*Cla002778*) in WT4 and WM102. The date represents the means ± standard deviations of three replicates.

To validate the BSA-seq results, 30 SSR markers and 30 indel markers in this region on chromosome 4 were selected for polymorphism screening between the two parental lines, WT4 and WM102. Six markers showed clear bands and good polymorphism, and they were further used for genotyping the F_2_ segregating population containing 1,106 plants. As a result, the *Clyr* gene was mapped between primer Indel590358 and the terminal of chromosome 4, covering a physical distance of 91.42 Kb (**Figure 11C**). According to the watermelon reference genome information, a total of three genes exist in the mapped region (**Figure 11C**). But expression analysis shows that expression level of the three genes are not obviously different between WT4 and WM102 (**Figure 12**).

## Discussion

Color is a focused trait of consumers for fruits and vegetables. Watermelon with yellow rind has become increasingly desirable for its delightful appearance and high carotenoid contents (Dou et al., 2018). Variation in rind color, including black, dark green, light green, and yellow are exhibited in watermelon (Guo et al., 2013). Chlorophylls and carotenoids are the main pigments influencing the color appearance of plants. According to the different content of these two pigments, the color of plants can vary from dark-green to yellow (Jabeen et al., 2017). In WT4, we found a dramatic reduction in chlorophyll a and chlorophyll b, but an increase in carotenoids, implying a close relationship between the yellow appearance and pigment content changes.

The key roles of metabolic pathways during rind color formation were studied with RNA-Seq technology to explore the transcriptomic differences between the two contrasting cultivated watermelon genotypes. A total of 581, 396, and 1,385 DEGs were obtained at 0, 7, 14 DAP. Because color of WT4 changes obvious for the three stages, the gene expression level of WT4 at 7 DAP and 14 DAP were also analyzed. Compared with the genes at 0 DAP, 4,010 DEGs were obtained at 7 DAP and 5,760 DEGs at 14 DAP were obtained compared with the genes at 7 DAP. The number of DEGs for WT4 at different stages was much greater than that of WT4 and WM102 in the same periods, implying a more active metabolism in the later stages and a group of genes that contribute to the development and color formation of these two watermelon cultivars. There were different DEG expression patterns in WT4. Some genes were up-regulated during the experimental stages, but some were down-regulated, and different patterns were also exhibited, suggesting a highly complex process concerning yellow rind color formation. Functional enrichment analysis of the DEGs was conducted to identify the most important pathways involved in rind color formation. In addition to the extensively enriched pathways in WT4 and WM102, some DEGs were found to be involved in chloroplast membrane, plant hormone signal transduction, photosynthesis, carbon fixation in photosynthetic organisms, and might have unique functions in pigment mechanism. These metabolic pathways are also important for color formation in apples and *Arabidopsis* (Miura et al., 2010; El-Sharkawy et al., 2015). In rice, the membrane localized short chain dehydrogenase encoded by gene *NYC1* represents a chlorophyll b reductase that is necessary for catalyzing the first step of chlorophyll b degradation. For the reason that chlorophyll b degradation was required for the degradation of light‐harvesting complex II and thylakoid grana in leaf senescence, the rice *nyc1* mutant shows the stay‐green phenotype (Sato et al., 2009).

Most of the typical color appearance in cucurbitaceous plants is controlled by a single dominant/recessive gene. In a previous research, the gene for watermelon yellow rind was mapped to a 59.8 Kb interval, but no target gene was found (Dou et al., 2018). But according to the newly released watermelon genome and primer information, the interval in the previous research should be 711.96 kb, not 59.8 kb. Bulked-segregant analysis (BSA) is an efficient method for screening markers tightly linked with the target genes for a given phenotype. It has been widely used for gene mapping study, but utilization of BSA methods requires DNA marker development and genotyping, which is time consuming and labor intensive. Next generation sequencing (NGS) technology could provide new ways to accelerate progress in gene mapping and isolation (Trick et al., 2012). With the BSA method, the gene *Clyr* was quickly mapped to the top end of chromosome 4. To further narrow down the mapped region, a large F_2_ population that provides more recombinants was analyzed with the polymorphic primers. There are three genes located in the mapped region. Gene *Cla97C04G068450* (*Cla002781*), *Cla97C04G068460* (*Cla002779*), and *Cla97C04G068470* (*Cla002778*) encodes the DNA glycosylase, the ATPase family AAA domain-containing protein, and DNA-binding storekeeper-related protein, respectively. The magnesium chelatase reaction is one of important step in chlorophyll biosynthesis pathways. The proteins BchI, BchD, and BchH are required to catalyze the insertion of Mg^2+^ into protoporphyrin IX upon ATP hydrolysis during the magnesium chelatase reaction (Willows et al., 1996; Hansson et al., 2013). AAA proteins are Mg^2+^-dependent ATPases, which usually play essential roles in proteolysis, membrane fusion, cytoskeletal regulation, protein folding, and DNA replication (Neuwald et al., 1999; Vale, 2000; Ogura and Wilkinson, 2001). Suppression of the ER-Localized NgCDC48, a member of the AAA ATPase superfamily, would make the leaves yellowish and inhibits tobacco growth and development (Bae et al., 2009). The above information suggests that gene *Cla97C04G068460* (*Cla002779*) can be the target gene. But the same expression level of *Cla97C04G068460* between WT4 and WM102 implicates that *Cla97C04G068460* may not be the target gene. And the unchanged nucleotide sequence of the cDNA, gDNA, and about 2,000bp bases before gene *Cla97C04G068460* in WT4 according to the resequecing result further confirmed that gene *Cla97C04G068460* would not be the gene for *Cl*yr. With GWAS analysis, three candidate genes associated with rind color and rind stripe were found on chromosomes 4, 6, and 8, corresponding to the rind trait loci S (foreground stripe pattern), D (depth of rind color), and Dgo (background rind color) (Park et al., 2016; Guo et al., 2019). Since *Dgo* gene (*Cla97C04G068530*/*Cla002769*) encodes a magnesium-chelatase subunit H involved in chlorophyll synthesis, which was not mapped in the candidate region, so the relationship between Dgo and yellow-rind trait in WT4 should be further studied.

In recent studies, a couple of transcription factors regulating plant rind color were identified. For example, a few of MYB-type transcription factors have been reported to affect plant pigment development and rind coloration in cucumber, sweet cherry, tomato, apple, and rice (Furukawa et al., 2007; Adato et al., 2009; Ballester et al., 2010; Li et al., 2013; Jin et al., 2016; Lun et al., 2016; Meng et al., 2016). Except the MYB-type transcription factors, many other transcription factors could determine plant coloration. Such as CsMADS6, a MADS transcription factor in sweet orange (*Citrus sinensis*), could modulate carotenoid metabolism by directly regulating carotenogenic genes (Lu et al., 2018). The F-box gene numbered *MELO3C011980* in melon was also speculated to negatively regulates flavonoid accumulation (Feder et al., 2015). In watermelon, a gene numbered *ClCG08G017810* that encodes a 2-phytyl-1,4-beta-naphthoquinone methyltransferase protein was speculated to be associated with formation of dark green rind color (Li et al., 2019). But according to another study, the transcription factor CmAPRR2 was identified as causative for the qualitative difference between dark and light green rind both in melon and watermelon (Oren et al., 2019). The transcription factors often participate pigment development and rind coloration in plant, but according to the watermelon genome information, no transcription factor was found in the mapped region, the gene for watermelon yellow rind may be not a transcription factor.

Non-protein-coding transcripts including small noncoding RNAs and long noncoding RNAs are reported to play pivotal roles in the epigenetic and post-transcriptional regulation of gene expression during growth, development, and stress response in plants. Xu found that the expression level of lncRNAs was tightly linked to DNA methylation and that endosperm hypomethylation could promote the expression of linked lncRNAs (Xu et al., 2018). In a dominant wax-less cabbage mutant, the target gene (*NWGL*) was confined to a region approximately 99-kb on the end of cabbage chromosome C08, but no DNA variance was found of the candidate gene (*Bol018504*) in this region. However, its reduced expression abundance and altered DNA methylation level was detected, which was speculated to be one of the possible reasons account for the mutant phenotype (Zhu et al., 2019). Considering the similar dominant mutant style and the no nucleotide changed in the target gene of *NWGL* gene, we predict that appearance of the yellow rind character in WT4 may also be the result of methylated modification. As how the regulators play its key roles during watermelon yellow rind formation, much more work needs to be done. Such as to further narrow down the mapped region and the functional study of the genes and the non-protein-coding transcripts in the mapped region.

## Data Availability Statement

The datasets generated for this study are located as follows: Genbank accession for the RNA-sequencing dataset of WT4 and WM102 is PRJNA549842. Genbank accession for the re-sequencing dataset of WT4 and WM102 is PRJNA551784. Genbank accession for the BSA analysis dataset of WT4 and WM102 is PRJNA576063.

## Author Contributions

LY and YY designed the study. MZ, DS, and XJW performed the RNA isolation and qRT-PCR experiments. DL and XCW performed the data analysis. HY and SY participated in the gene mapping and determination of pigment content. LY, HZ, DS, and DL wrote and revised the manuscript. All authors read and approved the final version of this manuscript.

## Funding

This work was financially supported by grants from the China Postdoctoral Science Foundation (2018M630823), National Natural Science Foundation of China (31902041), the Key Scientific Research Projects of the Higher Education Institutions of Henan Province (19A210002), and the Zhongyuan Youth Talent support program.

## Conflict of Interest

The authors declare that the research was conducted in the absence of any commercial or financial relationships that could be construed as a conflict of interest.

## Supplementary Material

Table S1 Sequences of primers for qPCR and gene mapping.

Table S2 Number of clean reads generated from each sample were sequenced and mapped to the 97103 genome.

Table S3 Sequences of the novel isoforms of unknown genes.

Table S4 Annotation of the novel genes to five protein databases.

Table S5 The correlation coefficients among three replicates of each period.

Table S6 Top 10 enrichment terms in biological process, cellular component, and molecular function in each group.

## References

[B1] Abe A. Kosugi S. Yoshida K. Natsume S. Takagi H. Kanzaki H. (2012). Genome sequencing reveals agronomically important loci in rice using MutMap. Nat. Biotechnol. 30 (2), 174–178. 10.1038/nbt.2095 22267009

[B2] Adato A. Mandel T. Mintz-Oron S. Venger I. Levy D. Yativ M. (2009). Fruit-surface flavonoid accumulation in tomato is controlled by a *SlMYB12*-regulated transcriptional network. PloS Genet. 5 (12), e1000777. 10.1371/journal.pgen.1000777 20019811 PMC2788616

[B3] Anders S. Pyl P. T. Huber W. (2015). HTSeq-a Python framework to work with high-throughput sequencing data. Bioinformatics 31 (2), 166–169. 10.1093/bioinformatics/btu638 25260700 PMC4287950

[B4] Andrews S. (2010). FastQC: a quality control tool for high throughput sequence data, Available online at: http://www.bioinformatics.babraham.ac.uk/projects/fastqc.

[B5] Bae H. Choi S. M. Yang S. W. Pai H. S. Kim W. T. (2009). Suppression of the ER-localized AAA ATPase NgCDC48 inhibits tobacco growth and development. Mol. Cells 28 (1), 57–65. 10.1007/s10059-009-0101-4 19711043

[B6] Ballester A. R. Molthoff J. de Vos R. te Lintel Hekkert B. Orzaez D. Fernández-Moreno J. (2010). Biochemical and molecular analysis of pink tomatoes: deregulated expression of the gene encoding transcription factor *SlMYB12* leads to pink tomato fruit color. Plant Physiol. 152 (1), 71–84. 10.1104/pp.109.147322 19906891 PMC2799347

[B7] Barham W. S. (1956). A study of the Royal Golden watermelon with emphasis on the inheritance of the chlorotic condition characteristic of this variety. Proc. Amer. Soc. Hort. Sci. 67, 487–489.

[B8] Bassi R. Dainese P. (1992). A supramolecular light-harvesting complex from chloroplast photosystem-II membranes. Eur. J. Biochem. 204 (1), 317–326. 10.1111/j.1432-1033.1992.tb16640.x 1740145

[B9] Benjamini Y. Hochberg Y. (2000). On the adaptive control of the false discovery rate in multiple testing with independent statistics. J. Educ. Behav. Stat. 25 (1), 60–83. 10.3102/10769986025001060

[B10] Boeckmann B. Bairoch A. Apweiler R. Blatter M. C. Estreicher A. Gasteiger E. A. (2003). The SWISS-PROT protein knowledgebase and its supplement TrEMBL in 2003. Nucleic Acids Res. 31 (1), 365–370. 10.1093/nar/gkg095 12520024 PMC165542

[B11] Britton G. Liaaen-Jensen S. Pfander H. (Eds.) (2008). Carotenoids, vol. 4: natural functions (Vol. 4) (Basel: Birkhäuser, Verlag: Springer Science & Business Media).

[B12] Cingolani P. Platts A. Wang L. L. Coon M. Nguyen T. Wang L. (2012). A program for annotating and predicting the effects of single nucleotide polymorphisms, SnpEff: SNPs in the genome of Drosophila melanogaster strain w1118; iso-2; iso-3. Fly> 6 (2), 80–92. 10.4161/fly.19695 22728672 PMC3679285

[B13] Dou J. Lu X. Ali A. Zhao S. Zhang L. He N. (2018). Genetic mapping reveals a marker for yellow skin in watermelon (*Citrullus lanatus L*.). PloS One 13 (9), e0200617. 10.1371/journal.pone.0200617 30265662 PMC6161839

[B14] El-Sharkawy I. Liang D. Xu K. (2015). Transcriptome analysis of an apple (*Malus* × *domestica*) yellow fruit somatic mutation identifies a gene network module highly associated with anthocyanin and epigenetic regulation. J. Exp. Bot. 66 (22), 7359–7376. 10.1093/jxb/erv433 26417021 PMC4765799

[B15] Feder A. Burger J. Gao S. Lewinsohn E. Katzir N. Schaffer A. A. (2015). A Kelch domain-containing F-Box coding gene negatively regulates flavonoid accumulation in muskmelon. Plant Physiol. 169 (3), 1714–1726. 10.1104/pp.15.01008 26358418 PMC4634078

[B16] Ferrer J. L. Austin M. B. Stewart C. Jr. Noel J. P. (2008). Structure and function of enzymes involved in the biosynthesis of phenylpropanoids. Plant Physiol. Bioch. 46 (3), 356–370. 10.1016/j.plaphy.2007.12.009 PMC2860624 18272377

[B17] Finn R. D. Bateman A. Clements J. Coggill P. Eberhardt R. Y. Eddy S. R. (2013). Pfam: the protein families database. Nucleic Acids Res. 42, D222–D230. 10.1093/nar/gkt1223 24288371 PMC3965110

[B18] Fromme P. Melkozernov A. Jordan P. Krauss N. (2003). Structure and function of photosystem I: interaction with its soluble electron carriers and external antenna systems. FEBS Lett. 555 (1), 40–44. 10.1016/S0014-5793(03)01124-4 14630316

[B19] Furukawa T. Maekawa M. Oki T. Suda I. Iida S. Shimada H. (2007). The *Rc* and *Rd* genes are involved in proanthocyanidin synthesis in rice pericarp. Plant J. 49 (1), 91–102. 10.1111/j.1365-313X.2006.02958.x 17163879

[B20] Grimm B. (1998). Novel insights in the control of tetrapyrrole metabolism of higher plants. Curr. Opin. Plant Biol. 1 (3), 245–250. 10.1016/S1369-5266(98)80112-X 10066589

[B21] Guo S. Zhang J. Sun H. Salse J. Lucas W. J. Zhang H. (2013). The draft genome of watermelon (*Citrullus lanatus*) and resequencing of 20 diverse accessions. Nat. Genet. 45 (1), 51–58. 10.1038/ng.2470 23179023

[B22] Guo S. Zhao S. Sun H. Wang X. Wu S. Lin T. (2019). Resequencing of 414 cultivated and wild watermelon accessions identifies selection for fruit quality traits. Nat. Genet. 51, 1616–1623. 10.1038/s41588-019-0518-4 31676863

[B23] Hörtensteiner S. (2006). Chlorophyll degradation during senescence. Annu. Rev. Plant Biol. 57, 55–77. 10.1146/annurev.arplant.57.032905.105212 16669755

[B24] Hansson M. Lundqvist J. Sirijovski N. Al-Karadaghi S. Subunits I. M. C. (2013). Magnesium chelatase: the molecular motor of chlorophyll biosynthesis. Handb. Porphyrin. Sci. 28, 41–84. 10.1142/9789814407755_0019

[B25] Harmut A. (1987). Chlorophylls and carotenoids: pigments of photosynthetic membranes. Method Enzymol. 148, 350–383. 10.1016/0076-6879(87)48036-1

[B26] Harris M. A. Clark J. Ireland A. Lomax J. Ashburner M. Foulger R. (2004). The Gene Ontology (GO) database and informatics resource. Nucleic Acids Res. 32, D258–D261. 10.1093/nar/gkh036 14681407 PMC308770

[B27] Hartigan J. A. Wong M. A. (1979). Algorithm AS 136: a k-means clustering algorithm. J. R. Stat. Soc. Ser. C (Applied Statistics). 28 (1), 100–108. 10.2307/2346830

[B28] Hill J. T. Demarest B. L. Bisgrove B. W. Gorsi B. Su Y. C. Yost H. J. (2013). MMAPPR: mutation mapping analysis pipeline for pooled RNA-seq. Genome Res. 23 (4), 687–697. 10.1101/gr.146936.112 23299975 PMC3613585

[B29] Hotta Y. Tanaka T. Takaoka H. Takeuchi Y. Konnai M. (1997). Promotive effects of 5-aminolevulinic acid on the yield of several crops. Plant Growth Regul. 22 (2), 109–114. 10.1023/A:1005883930727

[B30] Jabeen A. Kiran T. Subrahmanyam D. Lakshmi D. Bhagyanarayana G. Krishnaveni D. (2017). Variations in chlorophyll and carotenoid contents in Tungro infected rice plants. IBM J. Res. Dev. 5, 1–7. 10.4172/2311-3278.1000153

[B31] Jiang B. Liu W. Xie D. Peng Q. He X. Lin Y. E. (2015). High-density genetic map construction and gene mapping of pericarp color in wax gourd using specific-locus amplified fragment (SLAF) sequencing. BMC Genomics 16 (1), 1035. 10.1186/s12864-015-2220-y 26647294 PMC4673774

[B32] Jin W. Wang H. Li M. Wang J. Yang Y. Zhang X. (2016). The R2R3 MYB transcription factor *PavMYB 10.1* involves in anthocyanin biosynthesis and determines fruit skin colour in sweet cherry (P runus avium L.). Plant Biotechnol. J. 14 (11), 2120–2133. 10.1111/pbi.12568 27107393 PMC5095807

[B33] Kanehisa M. Goto S. (2000). KEGG: kyoto encyclopedia of genes and genomes. Nucleic Acids Res. 28 (1), 27–30. 10.1093/nar/28.1.27 10592173 PMC102409

[B34] Lange B. M. Ghassemian M. (2003). Genome organization in *Arabidopsis thaliana*: a survey for genes involved in isoprenoid and chlorophyll metabolism. Plant Mol. Biol. 51 (6), 925–948. 10.1023/A:1023005504702 12777052

[B35] Li H. Durbin R. (2009). Fast and accurate short read alignment with Burrows-Wheeler transform. Bioinformatics 25 (14), 1754–1760. 10.1093/bioinformatics/btp324 19451168 PMC2705234

[B36] Li Y. Wen C. Weng Y. (2013). Fine mapping of the pleiotropic locus *B* for black spine and orange mature fruit color in cucumber identifies a 50 kb region containing a R2R3-MYB transcription factor. Theor. Appl. Genet. 126 (8), 2187–2196. 10.1007/s00122-013-2128-3 23689749

[B37] Li B. Zhao S. Dou J. Ali A. Gebremeskel H. Gao L. (2019). Genetic mapping and development of molecular markers for a candidate gene locus controlling rind color in watermelon. Theor. Appl. Genet. 132 (10), 2741–2753. 10.1007/s00122-019-03384-3 31286160

[B38] Love M. I. Huber W. Anders S. (2014). Moderated estimation of fold change and dispersion for RNA-seq data with DESeq2. Genome Biol. 15 (12), 550. 10.1186/s13059-014-0550-8 25516281 PMC4302049

[B39] Lu S. Zhang Y. Zhu K. Yang W. Ye J. Chai L. (2018). The citrus transcription factor *CsMADS6* modulates carotenoid metabolism by directly regulating carotenogenic genes. Plant Physiol. 176 (4), 2657–2676. 10.1104/pp.17.01830 29463773 PMC5884614

[B40] Lun Y. Wang X. Zhang C. Yang L. Gao D. Chen H. (2016). A *CsYcf54* variant conferring light green coloration in cucumber. Euphytica 208 (3), 509–517. 10.1007/s10681-015-1592-z

[B41] Masuda T. Fujita Y. (2008). Regulation and evolution of chlorophyll metabolism. Photoch. Photobio. Sci. 7 (10), 1131–1149. 10.1039/b807210h 18846277

[B42] McKenna A. Hanna M. Banks E. Sivachenko A. Cibulskis K. Kernytsky A. (2010). The genome analysis toolkit: a MapReduce framework for analyzing next-generation DNA sequencing data. Genome Res. 20 (9), 1297–1303. 10.1101/gr.107524.110 20644199 PMC2928508

[B43] Meng R. Zhang J. An L. Zhang B. Jiang X. Yang Y. (2016). Expression profiling of several gene families involved in anthocyanin biosynthesis in apple (*Malus domestica Borkh*.) skin during fruit development. J. Plant Growth Regul. 35 (2), 449–464. 10.1007/s00344-015-9552-3

[B44] Miura E. Kato Y. Sakamoto W. (2010). Comparative transcriptome analysis of green/white variegated sectors in *Arabidopsis* yellow variegated2: responses to oxidative and other stresses in white sectors. J. Exp. Bot. 61 (9), 2433–2445. 10.1093/jxb/erq075 20400527 PMC2877895

[B45] Neuwald A. F. Aravind L. Spouge J. L. Koonin E. V. (1999). AAA+: A class of chaperone-like ATPases associated with the assembly, operation, and disassembly of protein complexes. Genome Res. 9 (1), 27–43. 10.1101/gr.9.1.27 9927482

[B46] Ogura T. Wilkinson A. J. (2001). AAA+ superfamily ATPases: common structure-diverse function. Genes Cells 6 (7), 575–597. 10.1046/j.1365-2443.2001.00447.x 11473577

[B47] Oren E. Tzuri G. Vexler L. Dafna A. Meir A. Saar U. (2019). Multi-allelic *APRR2* gene is associated with fruit pigment accumulation in melon and watermelon. J. Exp. Bot. 70, 3781–3794. 10.1093/jxb/erz182 31175368 PMC6685648

[B48] Park S. W. Kim K. T. Kang S. C. Yang H. B. (2016). Rapid and practical molecular marker development for rind traits in watermelon. Hortic. Environ. Biotech. 57 (4), 385–391. 10.1007/s13580-016-0005-0

[B49] Pruitt K. D. Tatusova T. Maglott D. R. (2005). NCBI Reference Sequence (RefSeq): a curated non-redundant sequence database of genomes, transcripts and proteins. Nucleic Acids Res. 33 (suppl_1), D501–D504. 10.1093/nar/gki025 15608248 PMC539979

[B50] Rüdiger W. (2002). Biosynthesis of chlorophyll b and the chlorophyll cycle. Photosynth. Res. 74 (2), 187–193. 10.1023/A:1020959610952 16228557

[B51] Rebeille F. Douce R. (2011). Biosynthesis of vitamins in plants part B. Adv. Bot. Res. 59, 2–292.

[B52] Roose J. L. Wegener K. M. Pakrasi H. B. (2007). The extrinsic proteins of photosystem II. Photosynth. Res. 92 (3), 369–387. 10.1007/s11120-006-9117-1 17200881

[B53] Saghai-Maroof M. A. Soliman K. M. Jorgensen R. A. Allard R. W. L. (1984). Ribosomal DNA spacer-length polymorphisms in barley: Mendelian inheritance, chromosomal location, and population dynamics. P Natl. Acad. Sci. U.S.A. 81 (24), 8014–8018. 10.1073/pnas.81.24.8014 PMC392284 6096873

[B54] Sato Y. Morita R. Katsuma S. Nishimura M. Tanaka A. Kusaba M. (2009). Two short-chain dehydrogenase/reductases, *NON-YELLOW COLORING 1* and *NYC1-LIKE*, are required for chlorophyll b and light-harvesting complex II degradation during senescence in rice. Plant J. 57 (1), 120–131. 10.1111/j.1365-313X.2008.03670.x 18778405

[B55] Schofield A. Paliyath G. (2005). Modulation of carotenoid biosynthesis during tomato fruit ripening through phytochrome regulation of phytoene synthase activity. Plant Physiol. Bioch. 43 (12), 1052–1060. 10.1016/j.plaphy.2005.10.006 16442806

[B56] Sundby C. Melis A. Mäenpää P. Andersson B. (1986). Temperature-dependent changes in the antenna size of Photosystem II. Reversible conversion of Photosystem IIα to Photosystem IIβ. BBA-Bioenergetics 851 (3), 475–483. 10.1016/0005-2728(86)90084-8

[B57] Takagi H. Abe A. Yoshida K. Kosugi S. Natsume S. Mitsuoka C. (2013). QTL-seq: rapid mapping of quantitative trait loci in rice by whole genome resequencing of DNA from two bulked populations. Plant J. 74 (1), 174–183. 10.1111/tpj.12105 23289725

[B58] Takaichi S. (2011). Carotenoids in algae: distributions, biosyntheses and functions. Mar Drugs 9 (6), 1101–1118. 10.3390/md9061101 21747749 PMC3131562

[B59] Takamiya K. I. Tsuchiya T. Ohta H. (2000). Degradation pathway (s) of chlorophyll: what has gene cloning revealed? Trends Plant Sci. 5 (10), 426–431. 10.1016/S1360-1385(00)01735-0 11044719

[B60] Tamiaki H. Shibata R. Mizoguchi T. (2007). The 17-propionate function of (bacterio) chlorophylls: biological implication of their long esterifying chains in photosynthetic systems. Photochem. Photobiol. 83 (1), 152–162. 10.1562/2006-02-27-IR-819 16776548

[B61] Trapnell C. Pachter L. Salzberg S. L. (2009). TopHat: discovering splice junctions with RNA-Seq. Bioinformatics 25 (9), 1105–1111. 10.1093/bioinformatics/btp120 19289445 PMC2672628

[B62] Trick,. M. Adamski,. N. M. Mugford,. S. G. Jiang,. C. C. Febrer,. M. Uauy,. C. (2012). Combining SNP discovery from next-generation sequencing data with bulked segregant analysis (BSA) to fine-map genes in polyploid wheat. BMC Plant Biol. 12 (1), 14. 10.1186/1471-2229-12-14 22280551 PMC3296661

[B63] Vale R. D. (2000). AAA proteins: lords of the ring. J. Cell Biol. 150 (1), F13–F20. 10.1083/jcb.150.1.F13 10893253 PMC2185557

[B64] Walker C. J. Weinstein J. D. (1994). The magnesium-insertion step of chlorophyll biosynthesis is a two-stage reaction. Biochem. J. 299 (1), 277–284. 10.1042/bj2990277 8166650 PMC1138051

[B65] Walvoort D. J. Brus D. J. De Gruijter J. J. (2010). An R package for spatial coverage sampling and random sampling from compact geographical strata by k-means. Comput. Geosci-UK. 36 (10), 1261–1267. 10.1016/j.cageo.2010.04.005

[B66] Willows R. D. Beale S. I. (1998). Heterologous expression of the Rhodobacter capsulatus BchI,-D, and-H genes that encode magnesium chelatase subunits and characterization of the reconstituted enzyme. J. Biol. Chem. 273 (51), 34206–34213. 10.1074/jbc.273.51.34206 9852082

[B67] Willows R. D. Gibson L. C. Kanangara C. G. Hunter C. N. Von Wettstein D. (1996). Three separate proteins constitute the magnesium chelatase of Rhodobacter sphaeroides. Eur. J. Biochem. 235 (1-2), 438–443. 10.1111/j.1432-1033.1996.00438.x 8631364

[B68] Xu W. Yang T. Wang B. Han B. Zhou H. Wang Y. (2018). Differential expression networks and inheritance patterns of long non-coding RNA s in castor bean seeds. Plant J. 95 (2), 324–340. 10.1111/tpj.13953 29738104

[B69] Young M. D. Wakefield M. J. Smyth G. K. Oshlack A. (2010). Gene ontology analysis for RNA-seq: accounting for selection bias. Genome Biol. 11, R14. 10.1186/gb-2010-11-2-r14 20132535 PMC2872874

[B70] Yuan H. Zhang J. Nageswaran D. Li L. (2015). Carotenoid metabolism and regulation in horticultural crops. Hortic. Res. 2, 15036. 10.1038/hortres.2015.36 26504578 PMC4591682

[B71] Zhu H. Song P. Koo D. H. Guo L. Li Y. Sun S. (2016). Genome wide characterization of simple sequence repeats in watermelon genome and their application in comparative mapping and genetic diversity analysis. BMC Genomics 17 (1), 557. 10.1186/s12864-016-2870-4 27495254 PMC4974753

[B72] Zhu X. Tai X. Ren Y. Chen J. Bo T. (2019). Genome-wide analysis of coding and long non-coding RNAs involved in cuticular wax biosynthesis in cabbage (Brassica oleracea L. var. Capitata). Int. J. Mol. Sci. 20, 2820. 10.3390/ijms20112820 PMC6600401 31185589

